# Molecular characterization of β-thalassemia intermedia in the West Bank, Palestine

**DOI:** 10.1186/s12878-019-0135-6

**Published:** 2019-02-18

**Authors:** Rashail Faraon, Mahmoud Daraghmah, Fekri Samarah, Mahmoud A. Srour

**Affiliations:** 1 Department of Medical Laboratory Sciences, Al-Quds University, East Jerusalem, Palestine; 2 Palestine Thalassemia Patients’ Friends Society, Al-Bireh, Palestine; 3 Department of Medical Laboratory Sciences, Arab-American University, Jenin, Palestine; 4 Department of Biology & Biochemistry, Birzeit University, P.O. Box 14, Birzeit, Palestine

**Keywords:** β-thalassemia intermedia, XmnI polymorphism, α-thalassemia, Palestine

## Abstract

**Background:**

We aimed to investigate the molecular basis of β-Thalassemia intermedia (TI) in the West Bank region and its management practices.

**Methods:**

This was a case series multi-center study and included 51 cases of TI. DNA sequencing was used to analyze β-globin gene mutations. Common α-globin gene mutations were screened by Gap-PCR (−α^3.7^, −α^4.2^, −-^MED^, ααα^anti3.7^) or DNA sequencing (α2-IVS II 5 nt del). *Xmn*I -158 C > T polymorphisms of Gγ-globin gene was determined by RFLP-PCR.

**Results:**

Seven β-globin gene mutations were observed, namely IVS-I -6 C > T, IVS-I-110 G > A, IVS-II-1 G > A, IVS-I-1 G > A, Codon 37 Trp > Stop, beta − 101 and IVS-II-848 C > A. Ten genotypes were observed. Homozygosity for IVS-I-6 accounted for the majority of TI cases with a frequency of 74.5%. The second common β-globin gene genotype was homozygote IVS-I-110 G > A (5.8%) and homozygote IVS-II-1 G > A (5.8%). The remaining seven genotypes were each detected in about 2% of patients. α-Thalassemia mutations were seen in five patients (9.8%), and included (−α^3.7^, ααα^anti3.7^ and α2-IVSII-5 nt del). *Xmn*I polymorphism was observed in four patients (7.8%), three homozygotes and one heterozygote.

**Conclusions:**

Homozygosity for the mild β-globin gene IVS-I-6 allele was the major contributing factor for the TI phenotype among the study subjects. The role of *Xmn*I SNP and α-thalassemia mutations in ameliorating the TI phenotype was observed in few patients for each factor. The beta − 101 C > T mutation was diagnosed in one patient in homozygote state for the first time in Palestine.

## Background

β-Thalassemia is one of the most common autosomal recessive disorders in the world as well as in Palestine. Up to date, more than 200 genetic mutations affecting the β-globin gene and associated with β-thalassemia have been reported [1], and of these mutations, 18 mutations have been reported among β-thalassemia patients from West Bank region of Palestine [2, 3]. β-Thalassemia intermedia (TI) is a heterogeneous group with a severity that is intermediate between the asymptomatic β-thalassemia trait (TT) and the transfusion-dependent β-thalassemia major (TM) [4]. TI and TM show overlap in their clinical symptoms and the differentiation between the two disorders shall enable TI patients to receive the proper clinical management and to avoid disease complications [5].

Despite the advances in the molecular genetics of TI, most cases of TI are still diagnosed based on clinical symptoms [6]. Genetically, TI results from mutations affecting primarily the β-globin gene production but coinheritance of other globin genes such as α- and γ-globin genes are also common. The molecular basis of TI phenotype can be attributed to three mechanisms: (i) inheritance of mild or silent β mutations, (ii) co-inheritance of α-thalassemia with β-thalassemia major, (iii) co-inheritance of determinants that increase γ-chain production [7–9]. The contribution of each of these mechanisms to the etiology of TI varies in different ethnic groups and their study is indispensable to adjust the management protocols for TI [5, 10, 11].

Few studies have determined the genetic mutations of TM and TT in the West Bank, but none had analyzed the molecular characterization of TI in the West Bank region, Palestine. So, this study aimed to determine the spectrum of genetic mutations associated with β-thalassemia intermedia as well as to investigate the hematological characteristics and assess the clinical management of TI patients in the West Bank region, Palestine.

## Methods

### Study design and patients

A case series retrospective multi-center study was conducted. All patients diagnosed with TI or met the inclusion criteria at the eight thalassemia centers in the major hospitals administered by the Palestinian Ministry of Health (MOH) in the West Bank were recalled. Samples were collected in the period November 2016 to October 2017. The inclusion criteria for TI were: (i) diagnosis of TI, (ii) age at diagnosis or initiation of transfusion ≥2 years, (iii) frequency of blood transfusion, once every 2–3 months or even longer period and (iv) questionable TM diagnosis such as a patient who is more than 30 years old or has high HbA2 levels. Based on these criteria, we have reviewed the medical files of all thalassemia patients and identified 70 potential TI patients from 436 thalassemia patients registered at all centers included in this study. All potential TI patients were contacted and 55 unrelated TI patients accepted to participate in this study. From these patients, 4 patients were found to have sickle β-thalassemia and thus were excluded from the study. The study protocol was approved by the Research Ethics Committee at Al-Quds University (document # 2/REC/2016). Also, permission from the MOH was obtained to review the medical files of patients at the thalassemia care centers. An informed consent was obtained from individual study participants or their guardians in case of minors.

Patients who accepted to participate in the study were asked to donate a 5 ml of blood. Most patients were met just prior to the next transfusion and those that have a long period between transfusions were asked to attend the thalassemia care center specifically to take part in this study.

### Clinical evaluation

Demographic and medical data about the enrolled patients were collected from the medical files using a questionnaire. Data collected included: patient’s age, sex, age on first blood transfusion, diagnosis, history of splenectomy / splenomegaly, the onset of disease, serum ferritin level, iron chelation therapy and any other relevant health complications. Additionally, one of the research team met the patients at the time of sample collection and asked him/her to verify the data collected from the medical files.

### Hematological analysis

Complete blood count (CBC) was done for all samples using automated hematology analyzer (Nihon Kohden). Hemoglobin electrophoresis was performed using HPLC method on the D-10 machine (Biorad).

### DNA analysis

DNA was extracted from EDTA whole blood for all samples using Genomic DNA Mini kit from Geneaid (USA). For analysis of β-globin gene mutations, the 5′ region of the gene was amplified using primer pair: Forward primer (F1) (5`-CGA TCT TCA ATA TGC TTA CCA A-3′) and Reverse primer (R1) (5’-CAT TCG TCT GTT TCC CAT TCT A-3′). The 3′ region of β-globin was amplified using the primer pair: Forward (F2) (5′- CAA TGT ATC ATG CCT CTT TGC A-3′) and Reverse (R2) (5′- TGC AGC CTC ACC TTC TTT CAT-3′). F1/R1 and F2/R2 primer pairs amplified 916-bp and 667-bp long amplicons, respectively, as described earlier [12]. The PCR amplicons were sequenced using standard Sanger DNA sequencing. DNA sequence results were analyzed visually and then using BLAST bioinformatics tool.

All patients were also screened for common α-globin gene mutations, namely –α^3.7^, −α^4.2^, and the –^MED^ and ααα^anti3.7^ by gap PCR, as described by Oron- Karni [13]. The α2- IVS2-5 nt del mutation was analyzed by DNA sequencing. For this purpose, the 5’region of α2-globin gene covering the mutation site was PCR amplified and sequenced as described earlier [9].

The XmnI SNP (− 158 C > T) at the 5’end of Gγ-globin gene was detected using RFLP-PCR using the primer pair: Forward primer (5′- AAC TGT TGC TTT ATA GGA TTT-3′) and Reverse primer (5′- TTT TAT TCT TCA TCC CTA GC-3′). The forward primer was described earlier by [14]. Digestion of the PCR product (592 bp) with XmnI allowed the differentiation of three possible genotypes: the T/T or −/− (592 bp); C/T or −/+ (592, 445, 147 bp); and C/C or +/+ genotype (445 and 147 bp).

### Statistical analysis

Descriptive statistics including mean and standard deviation were calculated using IBM SPSS version 23.

## Results

### Clinical findings

Fifty-one TI patients were enrolled in this study including 27 males and 24 females. The patients’ age ranged from 4 years to 71 years with a median of 18 years. The low age median of patients shows that this is a young population where 26 patients (50.9%) are below 18 years old. Among the 51 TI patients, 9 patients (17.6%) were never transfused, 9 patients (17.6%) received occasional transfusions (once or twice a year), while the remaining 33 patients (64.7%) were receiving regular transfusion (three to four times per year).

Analysis of the general health status of TI patients revealed that 19 patients (37.2%) were splenectomized, while 9 patients (17.6%) suffer from splenomegaly at time of enrollment. From the TI patients who were never transfused (*n* = 9), five patients were splenectomized; three patients were suffering from splenomegaly while one patient did not show symptoms of splenomegaly at time of enrollment in this study.

Thirty-one TI patients received iron chelation therapy for at least one year at the time of enrollment. Iron chelating drugs that were used by the TI patients included deferoxamine (Desferral) in eight patients, deferasirox (Exjade) in nineteen patients and four patients used a combination of the latter two drugs. Twenty TI patients did not use iron chelation therapy.

Most TI patients (92.2%) were the result of consanguineous marriages, as the parents of all patients were relatives except four patients (7.8%). None of the patients had ever leg ulcers, diabetes, hypertension (chronic disease) or documented venous thromboses.

### Hematological data

Hb levels ranged from 6.4 to 13.9 g/dL with a median of 8.3 g/dL at the time of enrollment. The serum ferritin levels were available for 31 patients and ranged from 92 to 8600 ng/mL with a median of 1800 ng/mL. In addition, two patients who were never transfused, had high serum ferritin (> 2700 ng/mL) and one patient was diagnosed at three years of age with the genotype β°/β°, has never been transfused and has an elevated level of serum ferritin (490 ng/mL). Twenty-two patients who had regular and occasional transfusion had high serum ferritin > 1000 ng/mL.

For hemoglobin electrophoresis patients were asked to donate blood samples for the study just before taking the next transfusion, mostly around 3 months after the last blood transfusion. HbF ranged between 1.3 and 83%, while HbA2 ranged between 2.3 and 8.5%. The level of HbF and HbA2 showed large variations reflecting the different genetic mutations responsible for the thalassemic phenotype.

### β-Thalassemia genotypes

A total of seven different mutations of the β-globin gene were detected and comprised null mutations (β^0^) as well as mild mutations (β^+^) allowing reduced synthesis of β-globin chain (Table 1). These seven β-globin gene mutations generated 10 genotypes that in turn were responsible for the phenotype of TI in our study subjects (Table 2). The most common allele of β-globin gene encountered was IVS-I-6 (T > C) with a frequency of 76.5% followed by IVS-I-110 (G > A) with a frequency of 7.8%, IVS –II-1 (G > A) with a frequency 6.9% and the remaining 4 alleles IVS-I-I, codon 37, IVS-II-848 and -101, accounted each for a frequency of 1–3% (Table 1). The most frequent single genotype was IVS-I-6 (T > C)/ IVS-I-6 (T > C) with a frequency of 74.5%, followed by IVS-II-1 (G > A)/ IVS-II-1 (G > A) with a frequency 5.8% and IVS-I-110 (G > A)/IVS-I-110 (G > A) with a frequency 5.8%, and the remaining seven genotypes each accounted for about 2% (Table 2).

**Table 1:** β-Globin gene mutations detected in Palestinian β-thalassemia intermedia patients. Allele frequencies were calculated based on 102 chromosomes from 51 patients

Mutation	Nt. Substitution	Type of mutation	HGVS nomenclature	Allele frequency (%)^a^
IVS-I-6	T > C	β^+^	HBB:c.92 + 6 T > C	76.5
IVS-I-1	G > A	β^0^	HBB:c.92 + 1G > A	2.0
IVS-I-110	G > A	β^+^	HBB:c.93-21G > A	7.8
Codon 37	TGG > TGA	β^0^	HBB:c.114G > A	3.0
IVS-II-1	G > A	β^0^	HBB:c.315 + 1G > A	6.9
IVS-II-848	C > A	β^+^	HBB:c.316-3C > A	1.0
Beta −101	C > T	β^++^	HBB:c.-151C > T	2.0

^a^The wild type allele (β^wt^) is present in one case from 51 patients and is not shown in the above table, making the sum of allele frequency 99.2 rather 100

**Table 2:** Hematological data and frequency of thalassemia genotypes among Palestinian β-thalassemia intermedia patients

β- thalassemia genotypes	N (%)	*Xmn*I SNP	α- thalassemia	Hb g/dL	Hb A2%	Hb F %
β^+^/β^+^						
IVS-I-6 / IVS-I-6	38 (74.5)	−/−	αα/ αα	8.4 ± 1.1	6.4 ± 1.4	10.2 ± 6.2
IVS-I-6 / IVS-I-110	1 (1.96)	−/−	α^IVSI(−5nt)^α/ αα	8.7	ND	ND
IVS-I-110 / IVS-I-110	3 (5.88)	−/−	α^3.7^/ αα (1:0:0)^a^	9/11.7/9.5	3.3/3.2/8.5	1.7/6.4/17
Beta −101/ Beta − 101	1 (1.96)	−/−	αα/αα	13.9	4.6	7.4
Subtotal	43 (84.3)					
β^+^/β°						
IVS-I-6 / Cd37	1 (1.96)	−/−	αα/ αα	6.9	3.3	6.6
IVS-I-110 / Cd37	1 (1.96)	−/−	αα/ αα	6.5	2.5	30.9
IVS-II-1 / IVS-II -848	1 (1.96)	+/−	–α^3.7^/ αα	7.1	3	49.1
Subtotal	3 (5.88)					
β°/β°						
IVS-II-1 / IVS-II-1	3 (5.88)	+/+	αα/ αα	8.7 ± 0.86	2.1 ± 0.23	72.7 ± 7.3
IVS-I-1 / Cd37	1 (1.96)	−/−	α^IVSI(−5nt)^α/ αα	8.7	2.9	46.8
Subtotal	4 (7.84)					
β^+^/β^wt^						
IVS-I-1 / β^wt^	1 (1.96)	−/−	αα/ ααα^anti3.7^	ND	3.8	6.3
Total	51 (100)					

*ND* not determined

^a^The numbers (1:0:0) are used to emphasize that only one patient (referred to as 1) has the α^3.7^/ αα genotype and his hematological values are placed at first position, respectively

To enable analysis of phenotype-genotype correlations, all the TI patients were grouped into four major genotypes (Table 2). The first one is the mild genotype β^+^/β^+^ which was further classified into four subgroups (Table 2).

Group I: Homozygosity for IVS-I-6 C > T. This group included 38 patients and showed a variable clinical picture with a mean Hb level of 8.4 g/dl. From 38 patients, 24 patients had a regular blood transfusion, 7 patients had an occasional transfusion, while the last seven had never been transfused. In this group, 15 patients were splenectomized while 6 patients suffer from splenomegaly.

Group II: Compound heterozygous for IVS-I-6 C > T / IVS-I-110 G > A with α^IVSI(−5nt)^α/ αα. This group included one patient and his Hb level was 8.7 g/dL. For this patient, Hb electrophoresis was not determined because he had blood transfusion one month before sample collection. This patient had a regular blood transfusion, was not splenectomized and showed no symptoms of splenomegaly at time of enrollment.

Group III: Homozygosity for IVS-I-110 G > A. This group included three patients. One of them had α^3.7^/αα genotype, had regular blood transfusion and showed splenomegaly. The other two patients had normal α-genotype, one of them had regular blood transfusion and showed no symptoms of splenomegaly while the second patient had occasional transfusion and was splenectomized.

Group IV: Homozygosity of the silent mutation beta − 101 C > T. This group included one patient and showed the highest Hb (13.9 g/dL) value among all study patients. The patient aged 13 years old and was never transfused.

Among all 4 subgroups, group I showed the highest percentage of HbF (10.2 ± 6.2%) (Table 2).

The second genotype β^+^/β° was classified into three different heterozygote subgroups, each containing one patient. Hb values ranged from 6.5 to 7.1 g/dL. One patient (β-globin genotype: IVS-II-1 G > A/ IVS-II -848 C > A) was heterozygote for both α-thalassemia (−α^3.7^/ αα) and ^G^γ-globin gene XmnI SNP and showed the highest percentage of HbF. All 3 patients with this β^+^\β° genotype had a regular blood transfusion.

The third genotype β^+^/β^wt^ was seen in one patient in association with α-globin gene triplication (αα/ααα^anti3.7^) and this patient had an occasional blood transfusion.

The fourth genotype was β°/β°. This group included four patients with Hb values ranging from 7.7 to 9.8 g/dL (Table 3). Usually, β-thalassemia patients with β°/β° genotype and normal α- globin gene show the TM phenotype. However, in this group the co-inheritance of Gγ-globin gene XmnI SNP in three patients (Table 3, patients # 1 to 3) and heterozygosity for α-thalassemia (α^IVSI(−5nt)^α/ αα) in one patient (Table 3, patient # 4) has ameliorated the thalassemic phenotype in this group. Patient # 3 (Table 3) has the highest HbF values, was never transfused and showed no symptoms of splenomegaly. While the other 3 patients (# 1, 2, 4; Table 3) were on regular transfusion (every 2–3 months), 2 patients (# 1 and 4) showed no symptoms of splenomegaly and one patient (# 2; Table 3) was splenectomized. This group also showed the highest percentage of HbF among the four groups listed in Table 2.

**Table 3:** Hematologic data and genetic modifiers for the 4 TI patients with β^0^/β^0^ genotype

Patient #	1	2	3	4
Age / age at diagnosis	17/6	16/5	29/3	39/1
Age at first transfusion, years	6	5	< 1	1
Hb, g/dL	7.7	8.6	9.8	8.7
MCV, fL	66.5	91.8	76.4	99.0
MCH, pg/cell	23.1	26.1	26.2	28.6
HbF, %	68.0	67.0	83.0	46.8
HbA2, %	2.3	1.8	2.3	2.9
β-thalassemia genotype	IVS-II-1/ IVS-II-1	IVS-II-1/ IVS-II-1	IVS-II-1/ IVS-II-1	IVS-I-1/ Cd37
*Xmn*I SNP γ^G^ − 158 C > T	+/+	+/+	+/+	−/−
α- thalassemia genotype	αα/αα	αα/αα	αα/αα	α^IVSI(−5nt)^α/ αα

Analysis of the effect of consanguineous marriages on β-thalassemia genotypes, revealed that among the 47 patients who belonged to relative parents, 44 patients had homozygote and 3 patients had compound heterozygote β-thalassemia genotypes. On contrast, of the 4 TI patients who belonged to non-relative parents, 3 patients had heterozygote and one patient had homozygote β-thalassemia genotypes.

### α-Thalassemia genotypes

From the 51 TI patients, 5 patients (9.8%) were found to have α-thalassemia mutations. Two patients were heterozygote for -α^3.7^/ mutation, two patients were heterozygote for α2-IVSII-5 nt del mutation and one patient was heterozygote for the ααα^anti 3.7^/ mutation. The -α^4.2^/ and --^MED^/ mutations were not detected in this study.

### XmnI polymorphism of the Gγ-globin gene

Out of 51 TI patients, four patients (7.8%) were positive for ^G^γ-globin gene *Xmn*I SNP (− 158 C > T, rs7482144). The three TI patients who were homozygote for the XmnI SNP showed the highest level of HbF compared to HbF percentage of all samples or that heterozygote for α-thalassemia (Table 4).

**Table 4:** Genetic modifiers of HbF level among TI patients. HbF percentages of all TI patients are compared with samples homozygote and heterozygote for *Xmn*I SNP, α-thalassemia or a combination of both

	All samples	*Xmn*I SNP (+/+)	α-thalassemia Heterozygotes	*Xmn*I SNP & α-thalass
N	51	3	5	1
HbF %				
Median	10.4	67.5	26.6	49.1
Range (min– max)	1.7–83.0	49.1–83.0	1.7–49.1	–
Hb g/dL				
Median	8.3	8.6	8.7	7.1
Range (min-Max)	6.4–13.9	7.7–9.8	7.1–9.0	–

One sample that was heterozygote for both XmnI SNP and α-thalassemia showed HbF percentage that is higher than the average of samples heterozygotes for α-thalassemia, but lower than that of samples homozygote for XmnI SNP (Table 4).

However, the limited number of samples positive for either XmnI SNP or α-thalassemia did not permit a reliable statistical comparison of means among these groups.

## Discussion

The study patients represent a young population with 50.9% of them are less than 18 years old. Analysis of the health data of TI patients showed that 64.7% of them had a regular blood transfusion and the median Hb was 8.3 g/dL. The median serum ferritin among the study patients was 1800 ng/mL and only 31 patients received iron chelation therapy. The decision to transfuse and the frequency of transfusion of TI patients in this study were based on their diagnosis as TI and on Hb level. However, the Thalassemia International Federation recommends that Hb level should not be an indicator for initiation of transfusion therapy for TI patients, except in patients with considerably severe anemia (Hb level < 5 g/dL) [15]. Thus, the transfusion frequency is inappropriate, at least in some patients, and should be reviewed case by case to determine the need as well as the frequency of transfusion. In addition, the high serum ferritin level in most patients suggests that their iron chelation therapy is inadequate. Previous studies showed that a better prognosis for survival was associated with a low serum ferritin level [16–18].

A total of seven different mutations of β-globin gene were detected and comprised null mutations (β^0^) as well as mild mutations (β^+^) allowing reduced synthesis of β-globin chain. The most common allele of β-globin gene encountered was IVS-I-6 (T > C) with a frequency of 76.5%. In consistency with our results, this allele was found to be the most frequent allele among Israeli Arabs with TI (i.e., Palestinians in Israel; (57.7%)) [19], as well as among β-thalassemia patients from West Bank region (28.7%) [2] and Southern region of West Bank (48.5%) [3] (Table 5). While the IVS-I-6 (T > C) allele was the third frequent allele among Palestinian β-thalassemia patients from Gaza strip [20]. The current study included only TI patients whose genotypes are mostly contributed by mild genotypes (β+) of β-globin gene. This, in turn, may partially explain the different frequencies of the IVS-I-6 (T > C) allele among our TI patients compared to the other two studies from the West Bank [2, 3] that used a mixed sample mostly comprised of TM patients. In addition, the IVS-I-6 (T > C) allele is a mild allele and is probably less frequent among TM patients compared to TI patients. In addition, the different frequencies of β-globin gene mutations among our TI patients compared to β-thalassemia patients from Gaza strip [20] reflect different genetic background of the Palestinian tribes in both regions of Palestine. In contrast, analysis of the frequency of IVS-I-6 (T > C) allele among β-thalassemia patients in neighboring Arab countries showed that this allele was ranked as the second, third, fifth or sixth common allele, among other β-globin gene alleles, in Iraqi Arabs [21], Egyptians [22], Lebanese [23], Jordanians [24] and Syrians [25], respectively (Table 5).

**Table 5:** The allele frequencies of β-thalassemia mutations in the current study compared to earlier studies from Palestine, other neighboring and some Middle Eastern countries

Nationality/ country	Palestinians				Israeli Arabs	Jordan	Syria	Lebanon	Egypt	Iraqi Arabs	Greece
Region	WB	WB	WB/s	Gaza							
Phenotype	TI	TM, TI	Mixed^a^	TM, TT	TI	Mixed ^a5^	TM, TT	Mixed^a^	TM	TI	TM, TI
No. of alleles analyzed	102	279	136	274	53	240	331	520	188	204	1179
Mutations											
IVSI-6 T > C	76.5	28.7	48.5	13.1	57.7	8.3	4.8	14.4	20.2	24	7.2
IVSI-110 G > A	7.8	17.6	9.5	33.9	8.5	25	15.7	34.2	57.4	11.3	42.5
IVSII-1 G > A	6.9	2.9	4.4	–	11.3	15	9.1	8.6	5.3	41.2	2
Cd37 TGG > TGA	3.0	10.4	11.3	2.6	1.4	6.3	2.1	–		0.5	–
IVSI-1 G > A	2.0	9.0	4.4	21.2	–	10	13.5	15	9.6	2	13.2
Beta −101 C > T	2.0	–	–	–	–	–	0.6	–	–	0.5	–
IVSII-848 C > A	1.0	2.5	–	–	–	1.3	0.6	–	–	1.5	–
Cd106/107 (+G)	–	6.8	–	–	–	–	–	–	–	–	–
Cd39 C > T	–	4.6	2.2	9.5	2.8	4.6	13.3	0.2	2.1	0.5	16.9
Cd5 (-CT)	–	2.5	8.1	0.4	–	3.8	4.5	5	–	0.5	1.12
Not-genotyped	–	8.2	3.7	–	5.6	1.6	7.8	0.2	–	–	–
Wild	1.0	–	–		4.2	–	–			1.5	–
Total No. of mutations	7	17	10	15	7	19	31	20	7	22	10
References	present study	[2]	[3]	[20]	[19]	[24]	[25]	[23]	[22]	[21]	[26]

WB: West Bank region; WB/s: southern part of WB. TI: β-thalassemia intermedia; TM: β-thalassemia major; TT: β-thalassemia trait

^a^Mixed: included TM, TI and TT and/or Sickle B-thalassemia but did not specify the allele frequency in each subgroup

Among our TI patients, 10 genotypes of β-globin gene were detected (Table 2). Of these genotypes, the most frequent single genotype was IVS-I-6 (T > C)/IVS-I-6 (T > C) and was encountered in 74.5% of TI patients. The next common genotypes were IVS-I-110/IVS-I-110 and IVS-II-1/IVS-II-1, where each was encountered in 5.88% of TI patients. The remaining seven genotypes were each detected in 2% of TI patients. These results indicate that the major contributing factor for TI among our study population is the inheritance of the mild β-globin gene allele (β+) and specially the IVS-I-6 (T > C) allele.

The second common mutation identified in this study was the Mediterranean IVS-I-110 G > A with an allele frequency of 7.8%. This allele was found to be the most frequent allele among β-thalassemia patients in Gaza strip (33.9%) [20]. In addition, earlier reports from Palestine, found that the IVS-I-110 G > A allele was the second frequent allele among β-thalassemia patients in West Bank (17.1%) [2], and the third frequent allele among β-thalassemia patients from Southern region of West Bank (9.5%) [3] (Table 5). In contrast, analysis of the frequency of IVS-I-110 G > A allele among β-thalassemia patients in neighboring Arab countries showed that this allele was ranked as the first common allele in Egyptians [22], Lebanese [23], Jordanians [24], Greece [26], and Syrians [25], while it is the third frequent allele in Iraqi Arabs [21].

The third frequent mutation was IVS-II-1 G > A with an allele frequency of 6.9%, which is considered as a severe allele (β^0^). Earlier reports from Palestine, reported this IVS II-1 G > A allele as the eighth and fifth frequent allele among β-thalassemia patients from West Bank (2.9%) [2], and from the Southern region of West Bank (4.4%) [3], respectively. However, this allele was not detected in Gaza strip [20]. It is the most common mutation in Iraqi Arabs 41.2% [21], and the second frequent allele in Israeli Arabs [19].

The next two alleles (IVS-I-1 and Codon 37) were found in our study at a lower frequency compared to previous reports from Palestine [2, 3, 20]. A previous study from Gaza Strip reported the IVS-I-1 allele as the most frequent allele and even showed a milder severity compared to the IVS-I-110 [27]. The allele (IVS-II-848 C > A) was reported earlier in West Bank [2] and at very low frequency among Jordanians [24], Syrians [25] and Iraqi Arabs [21].

The allele beta − 101 C > T was found at a low frequency in our study (2%) and this is the first report of this allele in Palestine. This allele was reported at a lower frequency in Syria [25] and Lebanon [23].

The combination of homozygous or compound heterozygous β-thalassemia with α-thalassemia decreases the excess alpha chains, and thus results in a less severe phenotype. α-Thalassemia mutations were seen in five patients only (9.8%) and in association with three β-globin gene genotypes: one β°/β^+^ plus –α^3.7^/αα and heterozygous state for the ^G^γ-globin gene *Xmn*I SNP; two β^+^/β^+^ (one with α2-IVSII-5 nt del and the other with -α^3.7^/αα); one β°/β° and α2-IVSII-5 nt del and one β^+^/β^wt^ plus α-triplication (αα/ααα^anti3.7^) (Table 3). Similar findings were reported from regional and neighboring Arab countries, where 7.8% (4/51) of Iraqi [4] and 9.8% (5/52) of Iranian TI patients [28] showed coinheritance of α-thalassemia mutations. The TI phenotype of another patient whose genotype was β^0^/β^0^ which is supposed to be severe is probably ameliorated by co-inheritance of at least two genetic modifiers, namely the existence of an α-thalassemia mutation (α^IVSI(−5nt)^α/ αα) and the existence of high HbF level (46.8%). In the latter case, the high levels of HbF are probably caused by genetic modifiers influencing HbF production [29, 30]. In the other two patients whose genotype was β^+^/β^+^ and co-inherited an additional α-thalassemia mutation, their Hb level is slightly above the average Hb of the study sample and thus the α-thalassemia mutation has probably slightly influenced their phenotype. Similar findings were reported in two of four members of a Jordanian family whose genotype was β^+^/β^+^ and –α^3.7^/αα, they were reported as non-transfusion dependent [31]. One patient was heterozygous for the β-globin gene (β^+^/β^wt^), but he co-inherited the α-triplication (αα/ααα^anti3.7^), which explains his TI phenotype. Since such alpha triplication increases the globin chain imbalance among β- thalassemia heterozygote and shifts the disease severity from TT toward the TI phenotype [8, 32, 33]. A similar finding in Israel was reported for a TT patient, who showed severe anemia and splenomegaly, although his genotype was β^+^/β^wt^ but he co-inherited the α-triplication αα/ααα^anti3.7^ [34].

Analysis of the Gγ-globin gene *Xmn*I SNP showed that three patients were homozygous and one patient was heterozygous for this SNP, three of them have the β^0^/β^0^ genotype and one has the β^0^/β^+^ (in addition to –α^3.7^/ αα) and the effect of this SNP was clearly illustrated by the high levels of HbF in these patients. The role of Gγ-globin gene *Xmn*I SNP in increasing HbF levels and moderation of thalassemia phenotype is widely known and reported in many earlier studies [4, 14, 35]. The *Xmn*I polymorphism is one of three major HbF quantitative trait loci (QTLs) responsible for HbF variation, and it leads to a less severe phenotype by increasing γ-chain production, which helps to neutralize unbounded α-chains [15, 36]. However, in the present study the ^G^γ-globin gene *Xmn*I polymorphism was not detected in any patient with the genotype β^+^/β^+^ or the β^+^/β^wt^. The latter findings are consistent with earlier reports that the *Xmn*I SNP is the commonest ameliorating factor in cases with β° mutations but not β^+^ [4, 37]. However, in our study the percentage of TI patients having the *Xmn*I SNP was low, in consistency with an earlier report from Brazil (9.7%) [38] but inconsistent to previous reports from Iraq (56.8%) [4], Iran (51.9%), [28] Pakistan (23%) [14] and China (26.5%) [39].

It is interesting that the three patients with the β^0^/β^0^ (IVS II-1/ IVS II-1) and homozygous for *Xmn*I SNP not only have a high level of HbF but also have Hb levels (mean ± SD = 8.7 ± 0.86) above the average levels of the study patients. A recent study on a cohort of Palestinian β-thalassemia patients from Gaza strip reported a milder phenotype for the homozygous IVS-I-1 allele and an association between this allele and the high expression level of HbF [27]. Ghoti el al. [27] found *Xmn*I SNP in only 7 out of 15 patients’ homozygotes for the IVS-I-1 allele, thus other genetic factors probably contribute to the high levels of HbF in association with null allele, which yet has to be determined. In addition, other studies have also reported an amelioration of the β^0^ allele by inheritance of the *Xmn*I SNP, including reports from Egypt, that showed that patients with the IVS-II-1 allele have relatively higher *Xmn*I SNP frequency (50%) than IVS-I-6 and IVS-I-110 [40], and a report from Southern Iran showed that 87.5% of patients with the IVS-II-1 allele were homozygous for the *Xmn*I SNP [41]. Thus, the low incidence of β° mutations among our study subjects may partially explain the low frequency of XmnI SNP detected in the present study.

The inheritance of the β ^+^ allele has been shown as the primary genetic modifier in TI in different reports [19, 26, 42] including the present study. The co-inheritance of α-thalassemia has been shown to be an important ameliorating factor in TI cases with either β^0^ allele [43] or β^+^ allele [42]. In addition, QTL affecting HbF expression (HBG2, BCL11A and HBS1L-MYB) have been shown to play an important role in ameliorating the thalassemic phenotype [21, 42–45]. In each of the three latter QTL, different SNPs have been studied and revealed different incidences among TI patients representing different ethnic groups [21, 42–45]. In a homogenous group of Sardinian β^0^-thalassemic patients, Galenello et al. [43] reported that all this group (50 cases of TI and 75 cases of TM) was negative for the XmnI SNP (HBG2) and their mild thalassemic phenotype could be mostly attributed to the co-inheritance of BCL11A, HBS1L-MYB SNPs as well as to α-thalassemia mutations. However, in most studies heterogenous groups of TI patients (associated with β^+^ and β^0^ alleles) were analyzed and the role of the three QTL were found to be variable [42–45] confirming the heterogeneity of TI and reflecting the different genetic background of each ethnic group studied.

In the present study, the frequency of β^+^ / β^+^ genotype is 84.3% (Table 2), and that of β^0^/ β^0^ genotype is 7.8% (Table 2). These findings indicate that the mild phenotype of TI in the majority of cases is due to inheritance of the β^+^ allele. While the co-inheritance of XmnI SNP and/or α-thalassemia could explain the mild phenotype of 5 TI cases from 7 cases associated with β^0^ allele. However, the presence of the other QTL (namely BCL11A and HBS1L-MYB) has not been determined yet.

Further studies with a larger number of patients and analysis of other QTL (BCL11A and HBS1L-MYB) may help reveal additional genetic modifiers contributing to the variations in HbF and milder phenotype associated with the β^0^ allele.

## Conclusions

This is the first study to report the molecular characterization of TI in Palestine. Genotyping of the β-globin gene detected 7 different mutations and 10 genotypes. The inheritance of the mild homozygote IVS-I-6 allele was the major contributing factor for the TI phenotype among study subjects. The beta − 101 C > T mutation was diagnosed in one patient in homozygote state for the first time in Palestine. The role of *Xmn*I SNP and α-thalassemia mutations in ameliorating the thalassemia phenotype was observed in few patients for each factor.

## Abbreviations

QTL: Quantitative trait loci
SNP: Single nucleotide polymorphism
TI: β-thalassemia intermedia
TM: β-thalassemia major
TT: β-thalassemia trait
